# Modulation of Fear Extinction by Stress, Stress Hormones and Estradiol: A Review

**DOI:** 10.3389/fnbeh.2015.00359

**Published:** 2016-01-26

**Authors:** Ursula Stockhorst, Martin I. Antov

**Affiliations:** Experimental Psychology II and Biological Psychology, Institute of Psychology, University of OsnabrückOsnabrück, Germany

**Keywords:** fear extinction, stress, monoamines, glucocorticoids, opioids, endocannabinoids, estradiol, exposure therapy

## Abstract

Fear acquisition and extinction are valid models for the etiology and treatment of anxiety, trauma- and stressor-related disorders. These disorders are assumed to involve aversive learning under acute and/or chronic stress. Importantly, fear conditioning and stress share common neuronal circuits. The stress response involves multiple changes interacting in a time-dependent manner: (a) the fast first-wave stress response [with central actions of noradrenaline, dopamine, serotonin, corticotropin-releasing hormone (CRH), plus increased sympathetic tone and peripheral catecholamine release] and (b) the second-wave stress response [with peripheral release of glucocorticoids (GCs) after activation of the hypothalamus-pituitary-adrenocortical (HPA) axis]. Control of fear during extinction is also sensitive to these stress-response mediators. In the present review, we will thus examine current animal and human data, addressing the role of stress and single stress-response mediators for successful acquisition, consolidation and recall of fear extinction. We report studies using pharmacological manipulations targeting a number of stress-related neurotransmitters and neuromodulators [monoamines, opioids, endocannabinoids (eCBs), neuropeptide Y, oxytocin, GCs] and behavioral stress induction. As anxiety, trauma- and stressor-related disorders are more common in women, recent research focuses on female sex hormones and identifies a potential role for estradiol in fear extinction. We will thus summarize animal and human data on the role of estradiol and explore possible interactions with stress or stress-response mediators in extinction. This also aims at identifying time-windows of enhanced (or reduced) sensitivity for fear extinction, and thus also for successful exposure therapy.

## Introduction

Classical fear conditioning (consisting of fear acquisition and extinction) is an influential experimental model to study emotional learning and memory. In fear acquisition, an initially neutral stimulus (cue or context) is paired with an aversive unconditioned stimulus (US) capable of eliciting a fear response (e.g., Phillips and LeDoux, 1992). After several pairings in the acquisition phase, the neutral stimulus becomes a conditioned stimulus (CS), now capable of eliciting a conditioned fear response (CR). Fear extinction is induced when the CS is repeatedly presented without the aversive outcome (US), resulting in a decline of CRs. In rodents, fear responses are typically assessed via freezing behavior (e.g., Bouton and Bolles, 1980; Fanselow and Helmstetter, 1988) or the fear-potentiated startle reflex (e.g., Brown et al., 1951; Davis, 1986). In humans, learning indicators used alone or combined include: skin conductance responses (SCRs), fear-potentiated startle, functional imaging data, as well as subjective ratings of US expectancy and/or CS valence/arousal (e.g., Grillon et al., 2004; Stark et al., 2006; Blechert et al., 2007; Milad et al., 2010; Soeter and Kindt, 2012; Bentz et al., 2013). Fear conditioning is highly adaptive, as it enables the organism to effectively and rapidly learn to predict danger using context information and environmental cues. However, fear learning can become dysfunctional if the organism continues to display fear responses in the absence of danger.

Both, fear acquisition and extinction, are valid to model features of anxiety disorders as well as trauma- and stressor-related disorders (such as posttraumatic stress disorder, PTSD). Specifically, *fear acquisition* can serve to model features of the etiology of these disorders. Correspondingly, simple phobias, social phobia, panic disorder, and PTSD are characterized primarily by dysregulated fear responses (Ehlers and Clark, 2000; Parsons and Ressler, 2013). Moreover, these disorders are characterized by deficits in *fear extinction*. This inability to inhibit fear responses is assumed to largely contribute to the maintenance of anxiety disorders (Lissek et al., 2005; Delgado et al., 2006; Mineka and Zinbarg, 2006; Mineka and Oehlberg, 2008), as well as trauma- and stressor-related disorders (e.g., Ehlers and Clark, 2000; Mineka and Oehlberg, 2008; Cover et al., 2014). PTSD is assumed to be related to and even caused by a failure to consolidate and retrieve memory for fear extinction (Quirk and Mueller, 2008). Correspondingly, patients with anxiety and especially trauma-related disorders show deficits in fear extinction learning and extinction recall (Lissek et al., 2005; Blechert et al., 2007; Michael et al., 2007; Milad et al., 2008, 2009b; Jovanovic et al., 2010; Glover et al., 2011; Norrholm et al., 2011; Inslicht et al., 2013). Moreover, *extinction learning* also serves as a model for exposure techniques in behavioral therapy (e.g., Milad et al., 2014).

The development of anxiety disorders and especially PTSD can be conceptualized as learning under severe stress. *Stress* is a state of actual or potential disruption in the individual’s internal/external environment registered by the brain and caused by factors we call *stressors* (Joëls and Baram, 2009). Stress leads to activation of the *stress response—*including activation of the central and peripheral nervous system and release of neuromodulators, hormones and transmitters—the *stress-response mediators—*in the brain and periphery. The stress response enables the organism to deal with the challenge by increasing central arousal, mobilizing energy, increasing cardiovascular tone, inhibiting costly processes such as reproduction, feeding, and digestion, and by modifying immune responses (Sapolsky et al., 2000; Chrousos, 2009). Thus, stress and the stress response are important for survival and are adaptive in nature. However, in some circumstances stress may cause pathology, as is the case in PTSD and other trauma- and stressor-related disorders. Traumatic situations not only include specific fear-related behaviors (e.g., flight, freezing) but also a significant amount of the less specific stress response. Thus, understanding how stress, the stress response, and specific stress-response mediators contribute to pathological changes seen in PTSD—such as impaired extinction—is of special importance. Moreover, understanding what conditions allow for normal functioning despite (traumatic) stress could advance our understanding of resilience and advance prevention. Last but not least, PTSD itself is accompanied by heightened stress (Maren and Holmes, 2016) and is also associated with changes of the stress system (e.g., Lupien et al., 2009). Both facts could interfere with the success of extinction-based exposure therapy. Consequently, examining effects of stress and single stress-response mediators on fear extinction could help improve treatment efficacy or even provide new targets for pharmacological treatment.

While anxiety disorders and trauma- and stressor-related disorders have a nearly twofold life-time prevalence in women as compared to men (Kessler et al., 1995, 2005; Tolin and Foa, 2006; Kilpatrick et al., 2013; for an overview, see Cover et al., 2014), there is increasing evidence for a role of the female sex hormone 17β-estradiol (E2) in these sex differences. Interestingly, there is first evidence that the quality of fear extinction is related to estrogen levels as supported by better extinction recall under high as compared to low E2-levels.

In line with the focus of the articles assembled in this Research Topic, we will concentrate on fear *extinction* and the role of stress and stress-response mediators in animals and in humans thereby also referring to the role of E2.

## Fear Acquisition and Fear Extinction

### Fear Acquisition and the Neuronal Fear Circuitry

The fear system can be conceptualized as an adaptive behavioral system that allows the organism to avoid, escape or face environmental threats (Rudy, 2014). The amygdala and its connections play a major role in the regulation of innate fear responses and in fear learning.

Fear acquisition involves an interplay between the basolateral amygdala (BLA), consisting of the lateral nucleus (LA), the basolateral and basomedial nuclei (together also referred to as basal nuclei or basal amygdala, BA), the central nucleus (CE), and the intercalated cell-masses (ITC), located between the BLA and the CE (LeDoux, 2007; Pape and Paré, 2010).

The LA serves as the primary input zone of the amygdala, receiving input from the auditory, visual, olfactory, somatosensory, and nociceptive systems (LeDoux, 2007; Pape and Paré, 2010; Herry and Johansen, 2014). In fear acquisition, information about the CS and the US converges into the LA. The LA is also a necessary site of synaptic plasticity underlying fear learning (LeDoux, 2007; Pape and Paré, 2010; Herry and Johansen, 2014; Tovote et al., 2015) and a main storage site for the fear memory trace (Pape and Paré, 2010).

The LA projects to the basal nuclei, and to the ITC. The ITC inhibit neurons in the CE (Royer and Paré, 2002) and thus prevent the defensive fear responses. The basal nuclei contain two types of neurons: so-called “fear neurons” and “extinction neurons” (Herry et al., 2008). Fear neurons fire when fear is expressed, and they maintain excitatory projections to neurons in the CE and in the prelimbic cortex. Extinction neurons, on the other hand, are active when fear has been extinguished and they project to the ITC (Rudy, 2014).

The CE is one main output region of the amygdala with projections to subcortical and brainstem areas. It coordinates defensive (fear) responses including freezing and endogenous opioid-mediated analgesia (periaqueductal gray, PAG), and startle reflex potentiation (nucleus reticularis pontis caudalis; Davis, 1992; Sah et al., 2003; Fanselow and Poulos, 2005; Pape and Paré, 2010). The CE is also connected to monoamine systems in the brain, including locus coeruleus (LC; noradrenaline, NA), dorsal/ventral striatum (dopamine, DA), and raphe nuclei [serotonin (5-hydroxytryptamine, 5-HT)]. These neuromodulatory connections enable the amygdala to influence the excitability of large portions of the brain, including many areas lacking a direct connection with the amygdala (Sah et al., 2003; Pape and Paré, 2010; Duvarci and Paré, 2014). Finally, the CE also activates hypothalamic nuclei producing the classical peripheral stress response with increased sympathetic arousal, hypothalamus-pituitary-adrenocortical (HPA) axis activation, and increased release of glucocorticoids (GCs) and adrenaline/NA into the bloodstream.

Thus, in the amygdala, fear expression is controlled by a fine interplay of inhibitory and excitatory microcircuits involving the BLA, CE, and ITC (Wolff et al., 2014; reviewed in: Duvarci and Paré, 2014; Dejean et al., 2015). The hippocampus is important for context fear conditioning and for encoding of contextual information in the conditioning situation and is thus assumed to contribute to the context-specificity of fear responses. Accordingly, hippocampus–amygdala interactions are regarded to be important for contextual modulation of fear (e.g., Tovote et al., 2015). Importantly, fear expression is also controlled by the medial prefrontal cortex (mPFC). Neurons in the dorsal part of the mPFC [the prelimbic cortex (PL) in rodents, and dorsal anterior cingulate cortex (dACC) in humans and other primates] promote fear expression (Likhtik and Paz, 2015) and both, BLA and PL/dACC activity, are modulated by the hippocampus (Herry and Johansen, 2014).

### Fear Extinction

While the fear system *per se* fulfills an adaptive function it can become maladaptive and produce pathologies such as anxiety, stressor- and trauma-related disorders (Rudy, 2014). Accordingly, conditions that improve the extinction of conditioned fear and hinder the return of fear are important in order to develop successful treatments of fear and anxiety disorders.

#### Stages of Fear Extinction Learning and Extinction Memory

In fear extinction, the CS is repeatedly presented without the US and CRs decline. As already suggested by Pavlov (1927), extinction learning does not destroy the original fear memory, but involves new learning. The organism acquires an inhibitory CS-no US association preventing the expression of fear responses (Bouton, 1994, 2002; Mueller and Cahill, 2010). This is supported by the observation that conditioned fear can easily reemerge even after successful extinction (Todd et al., 2014). This is the case when the US alone is presented after extinction *(reinstatement)*, when the CS is presented in a context different from the extinction context *(contextual renewal)*, or just with the passage of time *(spontaneous recovery)*.

**Figure 1:**
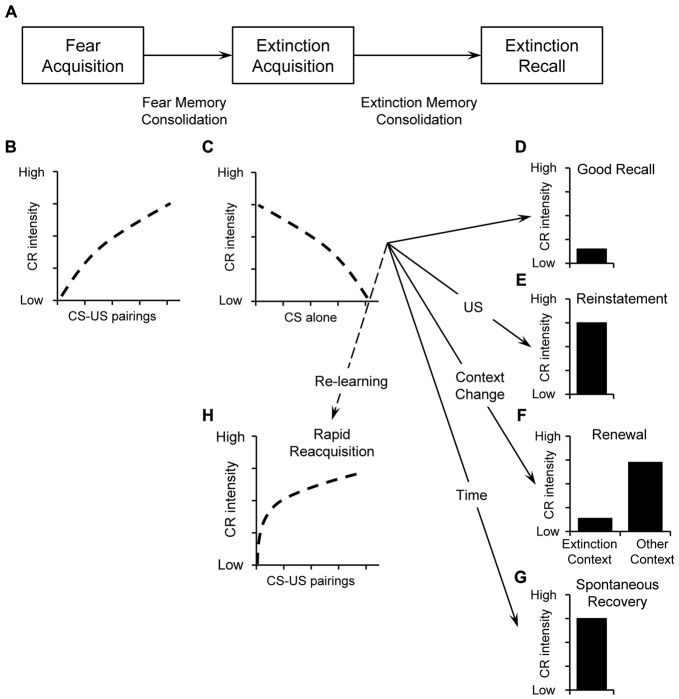
**(A)** Illustration of basic stages of fear and extinction learning and memory including acquisition, consolidation, and retrieval. During initial pairings between the conditioned stimulus (CS) and the unconditioned stimulus (US) during *fear acquisition*, responses increase over time **(B)**. Fear memory is consolidated thereafter. During *extinction acquisition*, the CS is repeatedly presented without the US and the conditioned responses (CR) decline with increasing number of CS alone presentations **(C)**. After consolidation, extinction recall can be tested by presenting the CS again. *Good extinction recall* is evident when CS-presentation triggers retrieval of the extinction memory trace and only low levels of CR occur **(D)**. Poor extinction recall—or return of fear—is evident in high conditioned responses despite successful extinction. Poor extinction recall can be caused by *reinstatement*, i.e., presenting the aversive US **(E)**, *renewal*, i.e., presenting the CS in a new context different from the extinction context **(F)**, or *spontaneous recovery*: i.e., by the passage of time **(G)**. Despite successful extinction acquisition, new CS-US pairing will result in faster reacquisition of the CR i.e., *rapid reacquisition* **(H)**.

Extinction covers three phases (Figure 1A): acquisition, consolidation and retrieval of extinction (Quirk and Mueller, 2008; Mueller and Cahill, 2010). Following fear acquisition (Figure 1B), extinction acquisition (Figure 1C) manifests as the decline of CRs during the initial extinction training session (Quirk and Mueller, 2008). Extinction can be quantified by the amount and speed of this decline. During consolidation, lasting at least several hours (Quirk and Mueller, 2008), cellular signaling cascades progressively stabilize the initially labile extinction memory trace into a consolidated memory (long-term memory; Baldi and Bucherelli, 2015). Extinction retrieval (or recall) is evident when subsequent presentation of the CS triggers retrieval of the extinction memory trace and only low levels of conditioned responding occur. Thus, good extinction recall will manifest in low CRs to the original CS (Figure 1D). Poor extinction recall—or *return of fear—*is evident in high CRs despite successful extinction. This is the case with *reinstatement* (Figure 1E), *renewal* (Figure 1F), and *spontaneous recovery* (Figure 1G). These return-of-fear phenomena are also a challenge for anxiety therapy, such as exposure therapies. Moreover, if the CS is paired with the US after extinction, we usually see a much faster reemergence of the CR (*rapid reacquisition*, Figure 1H).

Of note, the timing of different phases relative to each other is also important. For example, immediate extinction (minutes to hours after fear acquisition) vs. delayed extinction (24 h or more) were shown to produce different effects on return-of-fear phenomena. While in some studies immediate extinction produced less return of fear compared to delayed extinction (Myers et al., 2006), this was not replicated in other studies (Archbold et al., 2010; Golkar and Öhman, 2012). There is even evidence that immediate extinction produces poorer long-term fear reduction and more return of fear (Maren and Chang, 2006; Norrholm et al., 2008; Huff et al., 2009), i.e., an “immediate extinction deficit” (for a review, see Maren, 2014).

Importantly, stress, stress-response mediators and sex hormones may influence each of the phases of extinction learning and memory formation differently. Therefore, we will try to systematically review the available literature for effects on acquisition, consolidation, and retrieval of extinction. Where available, we will also report effects on return-of-fear phenomena and consider timing differences.

Presenting the CS without the US provokes a retrieval of the original fear memory. If the number of CS presentations is limited (1–4) this can trigger reconsolidation of the fear memory rather than acquisition of extinction. As originally described in the late 1960’s (Misanin et al., 1968), retrieval temporarily destabilizes the memory trace and makes it more susceptible to disruption. The reactivated memory is then actively re-stabilized (Nader et al., 2000). The increased plasticity is assumed to start about 10 min after retrieval and to last no more than 1 h (Johnson and Casey, 2015). This opens a brief reconsolidation-window during which pharmacological or behavioral intervention may erase the labile memory and prevent a return of fear. Blocking β-adrenergic receptors was found to disrupt reconsolidation in animals (e.g., Debiec and LeDoux, 2004, 2006; Rodriguez-Romaguera et al., 2009). There is also good evidence that β-adrenergic blockade could present a pharmacological tool in humans as well (Kindt et al., 2009; Soeter and Kindt, 2010, 2012; Sevenster et al., 2013; for a review, see Otis et al., 2015), but see also Bos et al. (2014). As a behavioral procedure, extinction training in the reconsolidation window was proposed and repeatedly shown to prevent the return of fear in humans in some studies (Schiller et al., 2010; Agren et al., 2012a) but not in others (Golkar et al., 2012; Kindt and Soeter, 2013). In general, blocking reconsolidation or using the reconsolidation window to modify traumatic memories is a promising technique that might reduce fear expression in patients with anxiety disorders or PTSD (for recent discussions, see Lane et al., 2015; Sandrini et al., 2015). A detailed review of reconsolidation studies is beyond the scope of our review. In the following sections, we will focus on effects of stress, and stress-response mediators on fear extinction. However, we will describe selected results from reconsolidation studies that also include measures of extinction learning and/or memory.

#### Brain Structures and Circuits Involved in Fear Extinction

Extinction, just as fear acquisition, is distributed over a network of structures, mainly covering the BLA, the hippocampus and parts of the mPFC, including the PL and the more ventral infralimbic cortex (IL) in rodents, and the dACC and ventromedial prefrontal cortex (vmPFC) as the corresponding structures in humans. Due to the underlying memory processes (consolidation, retrieval, reconsolidation) the involved neural circuits change over time (Maren and Holmes, 2016). While the same *structures* are involved in fear acquisition and fear extinction, *different sets of neurons* are assumed to act through different molecular mechanisms during fear acquisition and extinction (Maren and Holmes, 2016).

Besides fear acquisition, the amygdala is also involved in the acquisition, consolidation and retrieval of extinction (Quirk and Mueller, 2008). The hippocampus is the relevant site to recall contextual information. This becomes relevant because extinguished responses are often renewed in new contexts, differing from the original extinction context. Moreover, hippocampus and prefrontal cortex have a principal role in the regulation of the retrieval of both, fear acquisition and extinction memories (Fitzgerald et al., 2014). Concretely, the IL (in rodents) and the vmPFC (in humans) are vital structures (Tovote et al., 2015) for *extinction recall* (or *fear suppression*, Fitzgerald et al., 2014). The IL mPFC integrates CS-information with contextual information from the hippocampus to determine extinction retrieval. Thus, in the extinction context, the IL/vmPFC inhibits amygdala output to reduce the CR. The role of the IL specifically for extinction retrieval is under recent debate. A recent mice study by Do-Monte et al. (2015) showed that optogenetic silencing of glutamatergic IL neurons during cued extinction retrieval (1 day or 1 week after extinction acquisition) did not abolish retrieval. However, silencing IL neurons already during extinction acquisition impaired extinction retrieval on the following day. This supports the conclusion that—while relevant for the formation of extinction memory—the IL is *not necessary* for the retrieval of cued fear extinction. The one necessary structure for retrieval of cued extinction appears to be the amygdala. Moreover, these results are one example for recent data challenging the “canonical view” that dorsal regions (PL/dACC) of mPFC regulate fear expression and ventral regions (IL/vmPFC) fear suppression (for current reviews, see Likhtik and Paz, 2015; Giustino and Maren, 2015).

To conclude, extinction is assumed to involve functional changes in the network of amygdala, mPFC and hippocampus so that extinction networks inhibit fear networks (for an elegant review, see Tovote et al., 2015). For extinction to occur it is critical whether the CS activates the fear neurons or the extinction neurons in the BLA, whether ITC are activated, and whether there is an activation of specific subsets of neurons within the mPFC. Subsequently, when reporting effects of stress and different stress-response mediators on extinction we will consider data on direct effects in these brain structures where available.

## The Role of Stress and Stress-Response Mediators in Fear Acquisition and Extinction

### Relevance

Acquisition of anxiety disorders and especially of PTSD can be conceptualized as learning under severe stress. Enhanced acquisition of trauma-relevant fear is assumed to precede the development of PTSD (Bowers and Ressler, 2015). Accordingly, stress-enhanced classical fear conditioning is used as an animal model of PTSD (e.g., Rau et al., 2005). Importantly, stress and fear responses share common neural circuits, and the neuronal structures involved in fear acquisition and extinction are also highly sensitive to stress effects (e.g., Lupien et al., 2009).

Studying the effects of stress or stress-response mediators on fear acquisition and extinction thus constitutes a relevant paradigm to examine conditions that enhance or attenuate conditioned fear and fear extinction. This approach also aims at mimicking: (a) the stressful nature of the situation prevailing during the acquisition of the disorder and (b) the stressful nature of being re-exposed to the CS afterwards, e.g., during exposure therapy. Exposing subjects explicitly to additional stress or stress-response mediators within a fear-conditioning paradigm seems to be especially important in studies with humans, since the stimuli used as US are often considerably less stressful than US used in animal experiments. Despite the potential relevance, human studies on the effects of acute stress on fear extinction are still rare (for a review, see Raio and Phelps, 2015).

### Features and Mediators of the Stress Response

Stress is a process that involves multiple changes interacting in a time-dependent manner: with respect to their onset, these changes can be subdivided into a rapid first-wave and a (delayed) second-wave stress response (Sapolsky et al., 2000; Rodrigues et al., 2009). The neurotransmitters and brain areas involved in the stress response are at least partly identical with those involved in the fear circuitry.

**Figure 2:**
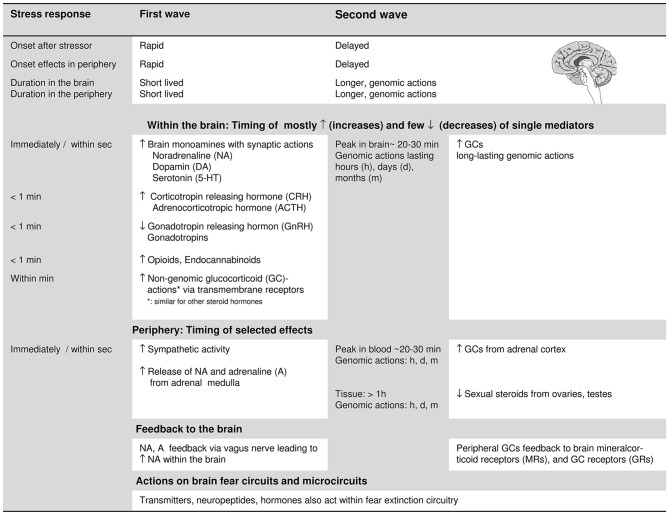
**Features of the rapid, first wave and the delayed second wave of the stress response: onset in brain and periphery and the timing of the activation of the mainly involved stress-response mediators (neurotransmitters, neuropeptides, hormones).** The concrete time specifications of the stress-induced changes are based on Sapolsky et al. (2000), Joëls and Baram (2009) and Hermans et al. (2014). For glucocorticoids (GCs) and sexual steroids, we differentiate between more rapid non-genomic, and delayed genomic actions. As to the stress-induced changes in the periphery, we only refer to the following, major changes: the rapid activation of the sympathetic nervous system and the corresponding rapid secretion of catecholamines, and to the increase in the secretion of GCs and the decrease in the secretion of sexual steroids from the adrenal cortex. The peripheral changes feedback to the brain and the single stress-response mediators also act on the fear extinction circuits and microcircuits (see “Contributions of specific stress mediators: insights from pharmacological manipulations, transmitter and neuropeptide actions” and “Insights from stress-induction studies triggering the first and second wave of the stress response” Sections). When referring to effects of single stress-response mediators on fear extinction within the text, we also take neuropeptide Y and oxytocin into account due to their role in stress resilience and their impact on fear extinction. The bodily reactions allowing adaptation to the stress challenge are not explicitly addressed in this figure.

The first-wave stress response (Figure 2) occurs within seconds and involves enhanced release of (a) monoamine neurotransmitters, i.e., of NA, DA, and 5-HT (Joëls and Baram, 2009), (b) corticotropin-releasing hormone (CRH) from the hypothalamus, followed by increased release of adrenocorticotropic hormone (ACTH) from the pituitary. Moreover, (c) there is a reduction in the release of gonadotropin-releasing hormone from the hypothalamus, and subsequently, a decrease in the secretion of gonadotropins, i.e., luteinizing hormone and follicle-stimulating hormone from the pituitary. Additional changes within the 1-min time frame (Sapolsky et al., 2000) involve the secretion of endogenous opiates, prolactin, glucagon, and growth hormone, as well as arginine-vasopressin and renin. In the periphery, the first-wave stress response includes a rapid increase in sympathetic tone and secretion of adrenaline and NA from the adrenal medulla (Sapolsky et al., 2000).

The second-wave stress response, starting after several minutes, involves (among others) increased peripheral secretion of GCs as a result of the activation of the HPA-axis, and reduced secretion of gonadal steroids (i.e., estrogens and gestagens in the ovaries, and testosterone in the testes; Sapolsky et al., 2000; Joëls and Baram, 2009). This inverse action on the secretion of peripheral GCs and gonadal steroids, and the interaction between HPA and hypothalamus—pituitary—gonadal axis under stress is of interest for the present review.

First- and second-wave changes not only have distinct temporal onsets but also differ in the onset and duration of their effects. In the brain, increased NA, DA, 5-HT, and CRH (acting on CRH-receptor 1) usually act within seconds but their effects subside quickly and rarely outlast the duration of the stressor; GC concentrations in the brain only reach peak levels about 20 min after stressor onset (Joëls and Baram, 2009; Hermans et al., 2014). Genomic actions of GCs (and other steroids) take even longer to manifest (Joëls and Baram, 2009). In fact, the slower genomic GC actions were proposed to actively reverse and normalize the rapid effects of various first-wave stress mediators (Hermans et al., 2014).

The brain also receives feedback from stress-induced changes in the periphery: peripheral catecholamines which cannot cross the blood-brain barrier, activate noradrenergic cells via binding to adrenergic receptors of the vagus nerve. The vagus nerve projects to the nucleus of the solitary tract. From there, projections reach the LC (Miyashita and Williams, 2002; Hassert et al., 2004). The amplified LC-activity increases NA levels throughout the brain, including the BLA (Rudy, 2014). Here, NA binds to adrenergic G-protein-coupled receptors and initiates the cAMP signal cascade (Rudy, 2014). Moreover, heightened central NA levels are related to an improvement in memory storage (Rudy, 2014). GCs readily cross the blood-brain barrier and exert negative feedback at hypothalamic and pituitary sites to reduce CRH and ACTH secretion (de Kloet et al., 2005). GCs also bind to higher-affinity mineralocorticoid receptors (MRs) and lower-affinity glucocorticoid receptors (GRs) throughout the brain, including the amygdala, hippocampus, mPFC, and septum (Joëls and Baram, 2009), where they can influence neural signaling and synaptic plasticity. Thus, they can also influence memory processes (Schwabe et al., 2012), such as those involved in fear extinction.

### Effects of Stress on Learning—Timing is Relevant

#### Overview

Acknowledging the fact that fear extinction covers encoding, consolidation and retrieval, models that studied the impact of stress on memory in other areas—such as declarative memory in humans and avoidance learning in rodents—should also provide predictions for fear extinction: these models reveal that the timing of the stressor relative to the learning task is important. Moreover, acknowledging the complexity of the multiple stress-response mediators over time, we will differentiate between mediators of the first wave and those of the second wave of the stress response.

#### The Role of Timing

Models derived from studies with inhibitory avoidance learning and object recognition (in animals) and declarative memory (in humans) suggest that stress effects on memory depend on the temporal proximity between learning and stressor: encoding close to the stressor (during the first wave of the stress response) will be enhanced, but encoding and recall later in time (during the second wave) will be suppressed (e.g., Schwabe et al., 2012).

*In detail.* Shortly after stress synergistic actions of rapid NA and non-genomic GC (as part of the first-wave stress response) in the BLA promote the *encoding* of emotionally relevant information by enhancing synaptic excitability and long term potentiation (Joëls et al., 2011), involving mainly glutamatergic actions. Slower genomic GC effects (as part of the second-wave stress response) will then enhance the consolidation of the material learned under stress. If however, the stressor is placed long before learning (i.e., stressor and learning are “out of sync”), stress should suppress the encoding of new material and impair the retrieval of previously learned material via second-wave stress mediators (such as genomic GCs).

There is evidence for the validity of these predictions from animal (Roozendaal et al., 2006b; Liebmann et al., 2009; Pu et al., 2009; Karst et al., 2010) and human studies (Henckens et al., 2010; Zoladz et al., 2011).

In animal pharmacological fear conditioning studies, reviewed by Rodrigues et al. (2009), evidence suggests that the first-wave stress mediator NA plays an important role in enhancing fear acquisition, whereas GCs as second-wave stress mediators enhance the consolidation, but have no effect on the acquisition of fear. The relevance of this model for fear conditioning in humans has to be tested.

## Modification of Fear Extinction by Stress-Response Mediators and Related Peptides

We will subsequently review the impact of stress on fear extinction (acquisition, consolidation, and retrieval). First, we will summarize data from pharmacological manipulations that use drugs that affect the brain’s monoaminergic, opioid and cannabinoid system, as well as CRH and GCs thus *specific mediators* involved in the first-wave and second-wave stress response. We here also include two peptides that might contribute to stress resilience (see “Further mediators: Neuropeptide Y [NPY] and Oxytocin” Section). Second, we will address *stress-induction studies* that trigger the first and second wave of the stress response (see “Insights from stress-induction studies triggering the first and second wave of the stress response” Section).

Modulation of fear extinction is also obtained via rapid glutamatergic and GABAergic transmission as well as cholinergic activity. Due to our focus on neurotransmitters and peptides that are involved in the stress response, we do not review these effects here. These systems are (only implicitly) included when we refer to the glutamatergic or GABAergic mediation of extinction processes.

### Contributions of Specific Stress Mediators: Insights from Pharmacological Manipulations, Transmitter and Neuropeptide Actions

#### Mediators of the First-Wave Stress Response

##### Monoamines

*Overview.* There is increasing evidence that the monoamines, i.e., NA, DA, and 5-HT, which are involved in the (first-wave) stress response (see “Features and mediators of the stress response” Section), also have roles in fear extinction: projections from noradrenergic, dopaminergic, and serotonergic fibers reach the main structures of the fear extinction network, the amygdala, the hippocampus, and the prefrontal cortex. Accordingly, administering agonists and/or antagonists of the respective monoamine transmitter in a fear extinction setting can elucidate the role of these monoamines. Moreover, experimental trials administering the respective drugs and testing their impact on extinction can serve as an experimental model for examining effects of the combination of drug therapy and behavioral exposure therapy (i.e., extinction learning). We will thus review animal data, preclinical data from healthy humans and clinical evidence from patient studies that address the therapeutic effects of the corresponding pharmacotherapy—also as an adjunct to cognitive behavioral therapy (CBT)—in anxiety disorders and/or PTSD (see also the excellent reviews of Fitzgerald et al., 2014; Bowers and Ressler, 2015; Singewald et al., 2015).

*Noradrenaline (NA).* NA was validated as a memory-enhancing transmitter in many studies on emotional memory, where β-adrenergic stimulation improved memory for emotional material (e.g., Cahill et al., 1994) interacting with arousal at encoding (Cahill and Alkire, 2003). Thus, it is reasonable that NA is also a key modulator of extinction learning and memory. A number of results support this:

The LC is the main localization of the cell bodies of NA-producing cells and innervates the neural structures of the extinction network, i.e., the amygdala, the hippocampus, and the prefrontal cortex (Mueller and Cahill, 2010). Mechanistically, NA acts to increase cellular excitability, and enhances synaptic plasticity within extinction-related circuits, and fear extinction was shown to require β-adrenergic activation in the IL-mPFC (Mueller and Cahill, 2010). Conditioned stimuli evoke NA release during extinction and strengthen extinction memory via β-adrenergic signaling (Mueller and Cahill, 2010). Moreover, GC-induced improvement of memory was shown to be mediated by β-adrenergic signaling (Quirarte et al., 1997; Roozendaal et al., 2002).

*Animals:* animal studies provide extensive evidence for *a role of central NA in the acquisition of fear extinction*. Selective forebrain NA-depletion prior to conditioning impaired fear extinction learning but not fear acquisition (Mason and Fibiger, 1979), suggesting that NA availability is needed for successful extinction learning. Lesions of the LC—the primer source of forebrain NA—also impaired extinction of the nictitating membrane reflex of the rabbit and the extinction deficit was positively correlated with the amount of NA depletion (McCormick and Thompson, 1982). Further support for a role of NA in enhancing extinction learning comes from studies with administration of the α2-receptor antagonist yohimbine, which leads to an increased presynaptic NA release. Yohimbine before extinction training reduced conditioned responses and enhanced the rate of fear extinction for cue (Cain et al., 2004; Mueller et al., 2009) and context fear conditioning (Cain et al., 2004).

A number of studies also suggest that *central NA may enhance the consolidation and/or the recall of extinction memory*: systemic yohimbine before extinction led to a superior extinction recall for cue (Cain et al., 2004; Morris and Bouton, 2007; Hefner et al., 2008) and context conditioning (Cain et al., 2004). However, yohimbine enhancement of fear extinction is context specific and does not prevent fear renewal (Morris and Bouton, 2007). Repeated systemic administration of the β-adrenergic agonist isoproterenol facilitated the consolidation of fear extinction, when administrated after each of three context exposure sessions (Do-Monte et al., 2010). A role for NA in consolidation processes of fear extinction is also supported by studies with central NA administration: NA-infusions into the right BLA, immediately (but not 3 h) after contextual fear extinction improved extinction recall at a delayed test, but NA without extinction training had no effect (Berlau and McGaugh, 2006). Other studies (Chai et al., 2014) suggest that the time window for NA effects on extinction might be different in different brain structures: Here, infusions into the CA1 area of the dorsal hippocampus improved long term extinction recall if NA was administered either immediately or 12 h after contextual fear extinction (Chai et al., 2014).

An increase of NA levels in the brain can be also achieved via electrical vagus nerve stimulation (VNS): in four experiments Peña et al. (2013) showed that VNS during CS-exposure accelerated extinction acquisition of both recent (24 h) and remote (2 weeks) fear memories. Consequently, VNS paired with the CS yielded better extinction recall compared to sham stimulation and unpaired VNS. In a newer study (Peña et al., 2014) VNS potentiated the IL-BLA pathway, thus contributing to enhanced extinction. Consistently, surgical vagal deafferentiation impaired cued fear extinction learning and was associated with decreased levels of NA and increased GABA-levels in the ventral PFC (Klarer et al., 2014).

For now, it is not entirely clear which adrenergic receptors mediate the effects of NA on extinction learning and memory. *Some studies suggest central β-adrenergic receptors*: repeated (but not single) systemic administration of the β-receptor blocker propranolol impaired both, the acquisition and the consolidation of contextual fear extinction (Do-Monte et al., 2010). Blocking β-adrenergic receptors in the IL PFC, impaired cued extinction recall without affecting responding during extinction learning (Mueller et al., 2008). Propranolol only impaired extinction recall when administered 10 min before extinction training, but not when administered immediately after extinction (Mueller et al., 2008), suggesting that β-adrenergic signaling in the IL during extinction training is needed to boost extinction memory formation. The delayed (12 h) enhancement of contextual extinction recall by NA in the CA1 region of the hippocampus (Chai et al., 2014) was also dependent on β-adrenergic receptors, activating the protein kinase A/cyclic adenosine monophosphate response element-binding protein (PKA/CREB) signaling pathway and increased GluR1 membrane trafficking. This supports a role for NA in the late stages of hippocampal memory formation. Central NA infusions also increased the excitability of IL pyramidal cells, and the effect was blocked by propranolol or Rp-cAMPS (a competitive antagonist of cAMP at the PKA binding site), suggesting that NA increases IL-excitability in a β-receptor and PKA dependent manner (Mueller et al., 2008).

*NA effects may also be co-mediated by α-adrenergic receptors:* the α1-adrenoceptor antagonist prazosin administered systemically after each extinction session over 6 days significantly slowed the reduction of conditioned freezing (Bernardi and Lattal, 2010). There is also some evidence *against* a role for central β-adrenergic receptors in NA-enhancement of fear extinction: systemic propranolol before extinction reduced CRs during extinction, but the reduction was significant from the first trial of extinction and there were no group differences at a recall test (Rodriguez-Romaguera et al., 2009). This is consistent with a reduction of fear expression (but not an enhancement of fear extinction). In the same study (Rodriguez-Romaguera et al., 2009), propranolol also reduced the firing rate of neurons in the PL mPFC, a region associated with fear expression (Sotres-Bayon et al., 2012; Courtin et al., 2014). One contextual fear conditioning study reports no effect on extinction recall in rats after bilateral BLA infusions of NA immediately after training (Fiorenza et al., 2012), and an improvement after the β-adrenoceptor antagonist timolol. In the same study (Fiorenza et al., 2012), bilateral infusions of NA into the vmPFC (incl. IL) impaired, whereas timolol improved contextual extinction recall.

*Humans:* there are only few studies investigating NA effects on fear extinction in humans. Healthy humans: in one study (Bos et al., 2012) propranolol administration before extinction impaired extinction learning in US-expectancy ratings, but not in SCRs or fear-potentiated startle. Reboxetine (a NA reuptake inhibitor, increasing central NA levels) administered after extinction acquisition had no effect on the consolidation of extinction memory (Lonsdorf et al., 2014).

Patients: in a sample of chronic PTSD-patients and traumatized controls, propranolol had no effect on delayed extinction recall when administered after extinction training (Orr et al., 2006). Finally, yohimbine was tested as adjunct to facilitate exposure therapy for anxiety disorders. Here yohimbine (vs. placebo) significantly improved the outcome of exposure therapy when combined with *in vivo* exposure for claustrophobia (Powers et al., 2009) and social anxiety disorder (Smits et al., 2014). Yet, in a virtual reality exposure therapy for specific phobia (fear of flying) yohimbine had no effect (Meyerbroeker et al., 2012).

*In sum:* animal data strongly suggest that NA can enhance extinction learning and extinction memory processes. Although NA effects were traced down to β-adrenergic signaling in extinction-relevant structures (BLA, IL-mPFC, hippocampus), the critical timing of effects and the relevant brain and molecular mechanisms are still a matter of debate, as is the contribution of other adrenergic receptor types (e.g., α1). In humans, there is very little data showing both, positive and negative results, which precludes clear conclusions. Given the strong animal evidence, more human studies on NA effects in extinction are warranted.

*Dopamine (DA)*. There is recent evidence that *long-term consolidation of extinction memory* involves dopaminergic signaling. The mPFC receives dopaminergic projections from the ventral tegmental area and these projections contribute to extinction via stimulating GABA-ergic cells in the amygdala (Fitzgerald et al., 2014). This should contribute to reduced amygdala activity and diminished expression of fear-related behavior. The role of DA (and also NA) for extinction is supported by animal data showing that cued fear extinction training in rats resulted in a concomitant increase of extracellular DA and NA in the mPFC (Hugues et al., 2007). In general, it is expected that extinction recall is improved by DA, but a number of procedural and pharmacological features are relevant.

The role of DA in extinction is typically examined by systemic administration of L-dopa (as the DA precursor), and DA agonists or antagonists, as outlined for some important animal studies and the available human studies.

*Animals and humans:* healthy individuals: Haaker et al. (2013) used L-dopa (vs. placebo) immediately *after extinction acquisition* in mice (three experiments) and humans (one experiment). To reveal DA’s role on *extinction consolidation*, return-of-fear phenomena were assessed. Mice: in a context-conditioning paradigm (experiment 1), L-dopa (as compared with placebo) led to lower spontaneous recovery 1 day, 7 days and 30 days after extinction acquisition (“extinction short”, 12 CS exposures), suggesting that L-dopa improves extinction recall. When the extinction phase was extended (“extinction long”, 30 CS exposures, experiment 2), spontaneous recovery (1 day and 38 days after extinction) was not diminished, but reinstatement (day 39) was significantly lower after L-dopa. Using cue conditioning, L-dopa (vs. placebo) resulted in less renewal (day 8) in the original acquisition context, a higher vmPFC-activity and lower amygdala-activity. These data further indicate that context-dependent extinction became context-independent via L-dopa. Humans: In the human study, cued fear acquisition and extinction acquisition were learned on the same day 1, followed by L-dopa (vs. placebo). One day later, L-dopa-treated subjects showed less behavioral renewal and a corresponding change in the neural fear circuitry with an increase in vmPFC- and a decrease in amygdala-activity. In a very recent human study (Haaker et al., 2015), with fear acquisition and extinction now conducted on separate days, post-extinction L-dopa only affected the neuronal indicators (downregulation of amygdala activity and upregulation of vmPFC activity) but not the behavioral level, i.e., SCR, during spontaneous recovery and reinstatement, now 8 days after extinction.

*Animals:* in line with the positive role of L-dopa for extinction memory, systemic administration of the NA- and DA-reuptake inhibitor methylphenidate (commonly prescribed for attention deficit hyperactivity disorder), enhanced extinction acquisition in a contextual fear paradigm when administered immediately before or after extinction training (Abraham et al., 2012). Extinction was tested for another three extinction sessions on separate days. Delivering methylphenidate 4 h after extinction training was not successful in improving extinction retention. Besides timing, substance type and dose have to be considered: Using amphetamine—which inhibits the reuptake of NA and DA non-selectively—prior to extinction, did not affect extinction acquisition of cued fear when ultra-low doses were used (Carmack et al., 2010), but was effective when given in higher doses (Mueller et al., 2009).

Considering the role of DA-receptor subtype actions, there was some initial evidence for a differential effect of D1- vs. D2-receptors: unexpectedly, antagonizing D2-receptors (via sulpiride), improved extinction memory whereas a D2 agonist (i.e., quinpirole), partially blocked extinction memory compared with the placebo controls (Ponnusamy et al., 2005). But a very well-controlled study (Mueller et al., 2010) meanwhile revealed that D2-receptor signaling is even necessary for extinction consolidation: the D2-antagonist raclopride which has a higher specificity and is more potent than sulpiride, was given systemically and locally (IL PFC) prior to extinction training. D2-antagonizing impaired not only extinction acquisition (day 2) but also extinction recall in a drug-free state (day 3). The authors even controlled for putative motor deficits provoked by D2-antagonists by circumventing its systemic action via direct administration into the IL PFC and found a reduced responsiveness of IL neurons to the CS after raclopride, assessed via single-unit recording in the IL PFC. This supports the interpretation that D2 receptors facilitate extinction by actions on IL PFC neurons that consolidate extinction.

Moreover, genetic differences in the dopamine transporter genes have to be taken into account (Agren et al., 2012b).

Interestingly, there is first evidence that estrogen is involved in modifying the efficacy of DA activity in the vmPFC, and thereby might affect extinction recall: D1-receptor activation in rats in a low estrogen status was able to rescue the extinction impairment found in these low-estrogen status rats (Rey et al., 2014). On the other hand, the initially better extinction recall in the high-estrogen rats diminished under the same D1-receptor agonist. These data reveal that the natural estrogen level and DA interact in a cycle-phase dependent manner and that a sex-hormone-related deficit can be compensated by actions of a D1-receptor agonist.

*In sum:* data support the idea that dopaminergic signaling does improve extinction memory, but that the drug’s dose, the temporal distance between acquisition and extinction, the duration of the initial extinction acquisition, the temporal distance between DA manipulation and testing, and female estrogen status has to be taken into account. DA-agonists might be useful as an adjunct to CBT.

*Serotonin (5-hydroxytryptamine, 5-HT).* Amygdala, hippocampus, and mPFC contain 5-HT and 5-HT receptors. Interestingly, fear conditioning *per se* (CS and US presentation) was shown to induce 5-HT increase: in cued fear conditioning, the aversive US led to an increase of extracellular 5-HT (and also DA and glutamate) in the BLA (Yokoyama et al., 2005). While the glutamate increase occurred only during acquisition (day 1), 5-HT and DA also increased as a response to the CS during extinction (day 2), although to a smaller amount and for a shorter duration (Yokoyama et al., 2005). Moreover, CS-exposure in fear-conditioned rats also induced a 5-HT release in the prefrontal cortex (Yoshioka et al., 1995). *In vitro* electrophysiological recording under administration of escitalopram, a selective serotonin reuptake inhibitor (SSRI), increased both, firing rate and bursts of dopaminergic neurons in the mPFC (Schilström et al., 2011).

The serotonergic system covers seven receptor families (5-HT_1_–5-HT_7_) with a total of 16 receptor subtypes with complex actions (Singewald et al., 2015). Research in fear extinction mainly addresses the role of three receptor subtypes: 5-HT_1a_, 5-HT_2_ and 5-HT_3_. 5-HT_1a_ and 5-HT_2_ receptors have been shown to be involved in the regulation of the excitability of the LA and the mPFC via activation of pyramidal cells and GABAergic interneurons (Singewald et al., 2015) and to contribute to an improvement of extinction memory. For 5-HT_3_ receptors, there is some evidence, that antagonists improve extinction (Singewald et al., 2015).

Studies addressing the role of 5-HT on fear extinction typically use SSRIs, SSNRIs (i.e., selective serotonin and noradrenaline reuptake inhibitors), or amphetamine, a less selectively-acting monoamine agonist that stimulates the release of all three monoamines, NA, DA, and 5-HT. Again, some exemplary animal studies and the available studies in humans are described.

*Animals:* in animals, chronic administration (21 days) of the SSRI fluoxetine (vs. placebo) after fear acquisition but *prior to extinction acquisition* was effective to improve extinction recall and to reduce return of fear (lower spontaneous recovery; Deschaux et al., 2011). Moreover, fluoxetine given *after extinction acquisition* prevented reemergence of fear when rats experienced a stressor (weak intensity shock) as a re-inducer of fear (Deschaux et al., 2013). Venlafaxine, a SSNRI, administered prior to extinction acquisition also improved extinction recall and diminished return of fear (Yang et al., 2012), while an effect on extinction acquisition was not found. This suggests a special impact of venlafaxine on extinction recall, not extinction acquisition. But substance-specific effects have to be taken into account: Chronic (22 days) administration of citalopram (which increases synaptic availability by binding to the 5-HT transporter protein) prior to extinction acquisition (Burghardt et al., 2013), resulted even in an impairment of extinction acquisition, accompanied by a downregulation of a specific subunit of the NMDA (N-methyl-D-aspartate)-receptor (NR2B) in the lateral and basal nuclei of the amygdala. Subchronic (9 days) administration did not induce this impairment.

SSRIs (although not all types) could be an important adjunct for success of exposure therapy, as evident in rats in a study comparing the effect of fluoxetine alone (vs. placebo), extinction training alone and the combination of both (Karpova et al., 2011): only the combination of chronic fluoxetine treatment (2 weeks after acquisition) and extinction training led to less return of fear, i.e., less renewal and reinstatement. On the neuronal level, fluoxetine increased synaptic plasticity in the BLA and the CA1 region of the hippocampus, related to a higher local action of brain-derived neurotrophic factor (BDNF). Thus, the authors conclude that erasure of fear needs “synergy” of pharmacological and behavioral treatment. However, one has to keep in mind, that fluoxetine was not only delivered prior to extinction and during extinction acquisition, but also during extinction recall testing. Thus, from this study, it remains unclear whether fluoxetine protection against return-of-fear phenomena (here: renewal, reinstatement) will also appear when being off the drug during recall testing. So far, effects might also be explained by state-dependency.

There is first evidence that sex and estrogen levels interact with the serotonergic system (Lebrón-Milad et al., 2013) in fear extinction: Chronic (14 days), not acute, fluoxetine following fear acquisition and preceding extinction acquisition improved extinction acquisition and extinction recall in female, not in male rats. In females, reduction of freezing was better when they were in a cycle phase with low as compared to high estrogen levels during extinction acquisition, suggesting that SSRIs might be helpful in women under cycle conditions of low estrogen levels where extinction recall is usually impaired (see “Sex, sex hormones and fear extinction” Section).

*Humans:* data in humans using fear acquisition and extinction measures is sparse so far. In healthy humans, 2 weeks of pretreatment with the very specific SSRI escitalopram (vs. placebo) prior to fear acquisition resulted in less SCR-responding to the CS+, i.e., improved extinction acquisition while fear acquisition was not modified (Bui et al., 2013).

Patients: there are several studies on the combinatory effect of serotonergic drugs and CBT, but the effects are validated by clinical ratings, not by fear conditioning (for reviews, see Bowers and Ressler, 2015; Singewald et al., 2015). So far, results are diverse, by either showing a positive effect of CBT only, of drug only, or a beneficial effect of the combination of both. But more data and especially experimental designs that really address both factors, drug and extinction (or exposure therapy) within the same study are needed.

*In sum:* the effects of serotonergic drugs on fear extinction vary with a number of factors: 5-HT receptor subtype, the actions of the drug on the presynaptic vs. postsynaptic binding sites (Bauer, 2015), acute vs. chronic administration, and the learning phase that is targeted (fear acquisition vs. fear extinction). Improvement of extinction (with extinction recall being more affected than extinction acquisition) was obtained mainly under chronic as compared to acute administration of 5-HT agonists given prior to extinction.

##### Opioids

Endogenous opioids (including endorphins, enkephalins, dynorphins and hemorphins; Singewald et al., 2015) are also involved in improving extinction learning (Quirk and Mueller, 2008). An increase of endogenous opioids is among the quick (<1 min) changes after stressor exposure (e.g., Sapolsky et al., 2000). Thus, a stress-mediated increase of opioids might participate in stress-mediated improvement of extinction, mainly via stressors affecting the first-wave stress response.

Opioid receptors (μ-, κ-, and δ receptors) are expressed in extinction-relevant brain areas, i.e., in amygdala, prefrontal cortices, and the hippocampus (for a review, see Singewald et al., 2015). Interestingly, ITC show a high expression of μ-opioid receptors (Blaesse et al., 2015), further suggesting that μ-opioid signaling might contribute to an improvement of fear extinction. The ventrolateral PAG (vlPAG) is a main site of the opioid actions during extinction learning, and vlPAG opioids are assumed necessary for extinction learning to occur. A very convincing interpretation for the involvement of opioids in extinction learning is that omission of an expected aversive US is rewarding (Quirk and Mueller, 2008).

Studies addressing the role of opioids for fear mainly rely on the administration of opioid antagonists, as illustrated by a few animal studies and studies in humans.

*Animals:* in rats, the administration of the μ-receptor opioid antagonist naloxone impaired within-session extinction of fear, while the impairment did not occur when naloxone was administered after extinction training (McNally and Westbrook, 2003). Facilitation of fear extinction was obtained by within-vlPAG-infusion of RB101(S), a drug that inhibits encephalin degrading enzyme, but not when infused outside the vlPAG (McNally, 2005).

Interestingly, there are interactions between morphine and female sex-hormone levels in fear extinction (Perez-Torres et al., 2015): subcutaneous morphine injections immediately after fear acquisition (day 1) led to impaired extinction acquisition (day 2) when female rats were in a cycle phase of low estradiol and low progesterone (P4) level (metestrus)—as compared to females with high levels of ovarian hormones and males. Moreover, in the metestrus group, there was no increase of μ-opioid receptor expression in the amygdala. Group differences were not found when morphine was injected only at the next day (day 2), 4 h prior to extinction. Thus, in a state of low-level ovarian hormones, morphine administered immediately after a trauma (fear acquisition) might unintendedly even increase the recall of fear.

*Humans:* in humans, extinction deficits were found under genetic variations of opioid-receptor expression: subjects with a single nucleotide polymorphism in the gene encoding prodynorphin (acting on the κ-opioid receptor), exhibited impaired fear extinction and reduced functional connectivity between amygdala and vmPFC (Bilkei-Gorzo et al., 2012).

Patients: Arntz et al. (1993) examined the effect of the blockade of opioid transmission via the opioid antagonist naltrexone in 48 spider-phobics that underwent a 2 h exposure session given low-dose, or high-dose naltrexone, or placebo. Behavioral avoidance measures, emotional, physiological and cognitive measures were assessed prior to, during, and one week after exposure. Opioid blockade resulted in a dose-related relapse of behavioral avoidance while the other measures did not differ. Morphine was also found to be important for the secondary prevention of PTSD (for a review, see Bowers and Ressler, 2015): children traumatized by acute burns developed less PTSD symptoms when given morphine post trauma (e.g., Stoddard et al., 2009). Moreover, the morphine dose in the 48 h after trauma was one of the predictors of PTSD severity 3 months after the trauma: patients with the later diagnosis PTSD had obtained less morphine in the aftermath of the traumatic injury (Bryant et al., 2009). Based on the data from Perez-Torres et al. (2015), estradiol levels should be taken into account and be validated for PTSD immediately after trauma exposure.

*In sum:* data converge to reveal an impairment of extinction via opioid antagonists and evidence for an improvement of symptoms in trauma-related disorders when morphine is given after the trauma. But the exact timing of opiate administration and/or of opioid action as well as the cycle phase in females has to be taken into account. With regard to its pain-inducing effects, the cold pressor test (CPT) used as a means to induce acute stress in fear conditioning studies in humans (see “Insights from stress-induction studies triggering the first and second wave of the stress response” Section), is a very interesting candidate because it does not only provoke a rapid increase of NA, but should also affect the opioid system.

##### Cannabinoids

Cannabinoid-type 1 (CB1) receptors have a high density in amygdala and hippocampus. The endogenous production of cannabinoids via the endocannabinoid system is regarded to be relevant for emotional and cognitive processing of threatening stimuli (Bitencourt et al., 2008). As recently reviewed (Quirk and Mueller, 2008; Fitzgerald et al., 2015; Papini et al., 2015), there is evidence for a facilitating role of endocannabinoid signaling in extinction learning via the CB1-receptor. A role of endocannabinoids (eCBs) for improving fear extinction is also supported by increased levels of eCBs in the BLA after extinction training (Marsicano et al., 2002). Moreover, eCBs and CB1-receptors were shown to induce depression of GABA-mediated inhibitory currents (Marsicano et al., 2002).

Importantly, stress and GCs influence eCBs levels (reviewed in Maren and Holmes, 2016): stress and GCs increase eCBs in the BLA, and eCBs act on brain GCs and on the HPA axis (Hill et al., 2010). eCBs were also shown to contribute to an NA-induced improvement of extinction *memory* by the following GC-related actions: An increase of GCs (induced in an emotionally aversive situation) at the brain GRs was shown to activate pathways that induce eCB synthesis. eCBs then inhibit GABAergic neurons, and thus disinhibit NA release (Atsak et al., 2012)—or in other words, increase NA recruitment. Thus, eCBs might contribute to an NA-mediated improvement of extinction memory after preceding GC increase.

The role of eCBs in fear extinction is typically examined using transgenic mice lacking the CB1-receptors, or by administering CB1 agonists and antagonists or inhibitors of enzymes that are involved in the reuptake or breakdown of eCBs (for a review, see Papini et al., 2015).

*Animals:* CB1-knockout mice show normal acquisition of fear conditioning (freezing behavior), but impairment of extinction acquisition and extinction retention suggesting a role of CB1 for extinction, not acquisition of fear (Marsicano et al., 2002). Accordingly, blockade of the CB1 receptor was shown to impair extinction learning, while administration of CB1 agonists and CB reuptake inhibitors improved extinction in animals in a number of studies as specified below:

As to CB1-antagonists, SR141716A (Marsicano et al., 2002) impaired extinction acquisition and recall, but not fear acquisition. Similarly, the CB1-antagonist rimonabant dose-dependently reduced extinction learning of fear-potentiated startle (Chhatwal et al., 2005). Correspondingly, CB1 agonists improved extinction recall: the CB1-agonist phytocannabinoid cannabidiol (Bitencourt et al., 2008), and AM404, an eCB reuptake inhibitor injected *prior to* *extinction acquisition*, facilitated extinction recall (Chhatwal et al., 2005; Bitencourt et al., 2008) and reduced reinstatement (Chhatwal et al., 2005). AM-3506 (preventing the degradation of anandamide), given either systemically or infused locally into the amygdala prior to extinction, also resulted in better extinction recall, while it had no effect in the absence of extinction training (Gunduz-Cinar et al., 2013).

Evidence for the local action of eCB in fear extinction circuitry is further supported by results that pre-extinction infusions of CB antagonists into fear-relevant brain areas impaired extinction, while pre-extinction infusion of agonists improved extinction recall, but results are still mixed (Papini et al., 2015).

*Humans:* Klumpers et al. (2012) compared effects of Δ-9-tetrahydro-cannabinol (THC) vs. placebo administered *prior to* *extinction acquisition* in healthy humans: they found an improvement of extinction acquisition (lower number of CRs) in SCRs, but not in fear-potentiated startle; the improvement did no longer remain in the retention test. But effects on extinction recall were found in other studies (Das et al., 2013; Rabinak et al., 2013, 2014): when administering cannabidiol, a CB1-receptor agonist and non-psychoactive component of cannabis (vs. placebo), either prior to or following extinction acquisition (Das et al., 2013), extinction acquisition was not affected, but extinction recall improved (manifesting in expectancy ratings) when cannabidiol had been administered after extinction acquisition, thus suggesting an effect on consolidation of extinction memory. Less reinstatement (trend level in the SCRs) was found after both time-points of eCB-administration, prior and after extinction training. Longer-lasting effects on extinction recall (day 3) were also observed after a single oral dose of the synthetic form of Δ-9-THC, dronabinol (vs. placebo) administered prior to (not after) the extinction acquisition (day 2; Rabinak et al., 2013). In a similar study, another synthetic THC, marinol, now did not affect SCR during extinction recall (day 3) but led to an increase of vmPFC and hippocampus activation to a previously extinguished CS+ in a differential conditioning paradigm (Rabinak et al., 2014).

Probably, genetic markers might help to reconcile the partly inconsistent results: healthy humans with reduced CB1-receptor expression showed impaired fear extinction in a virtual reality paradigm (Heitland et al., 2012).

Patients: the contribution of impaired eCB-signaling in patients with PTSD is also a matter of debate and is elegantly reviewed by Papini et al. (2015). In a recent study using positron emission tomography (PET), patients with PTSD were shown to have a ~50% reduction of the peripheral eCB anandamide and a ~20% higher availability (upregulation) of CB1 receptors in amygdala, hippocampus, and the cortico-striatal network than healthy controls. This pattern was more pronounced in women (Neumeister et al., 2013). Thus, a lower anandamide-tone might contribute to symptoms of PTSD and might lead to impaired extinction.

*In sum:* there is evidence for a positive role of eCBs for enhancing extinction recall and—correspondingly—for an impairment of extinction recall when eCB signaling is impaired. Moreover, acute, not chronic drug administration appears to be helpful to improve extinction recall. Further, more studies have to be conducted to find out how low eCB levels and upregulation of brain CB1-receptors might contribute to PTSD. The interaction between eCBs and stress is very interesting in the context of this review.

##### Corticotropin releasing hormone (CRH)

CRH neurons and CRH receptors are not only available in stress-responsive areas, but also in areas of the fear circuitry, i.e., in the BLA and the CE of the amygdala (Gafford and Ressler, 2015).

*Animals:* animal data show that intra-BLA infusion of CRH and of CRH binding protein (leading to an increase of endogenous CRH levels) prior to fear extinction impaired extinction recall while not affecting extinction acquisition. Correspondingly, a CRH-receptor antagonist improved extinction recall (Abiri et al., 2014). Similarly, in a transgenic mouse model missing the GABA(A)α1 subunit of the CRH neuron, CRH messenger RNA (mRNA) was elevated in the amygdala, the BNST and the hypothalamic paraventricular nucleus. The transgenic mice showed impaired extinction of conditioned fear and higher plasma cortisol levels. Deficits were successfully treated by systemic as well as local (BNST) infusion of a CRH-antagonist (Gafford et al., 2012).

*Humans:* in patients with PTSD, a hyperarousal of CRH pathways is described (Gafford and Ressler, 2015). Currently, a multicenter randomized controlled trial is in progress testing the effects of a CRH-type 1 receptor antagonist (GSK561679) in women with PTSD (Dunlop et al., 2014). Women will receive either 6 weeks drug treatment or placebo, and among a large number of dependent variables, fear acquisition and extinction will be assessed.

#### Further Mediators: Neuropeptide Y (NPY) and Oxytocin

Recent data also provide a role for NPY and oxytocin in extinction memory. Both peptides are known for being involved in stress resilience.

*Neuropeptide Y.* High concentrations of NPY are found in the cortex, amygdala, the hippocampus and the PAG, and in hypothalamic regions. NPY acts through six receptor subtypes, (Y1 to Y6), with Y1, Y2, Y4 and Y5 as the functional subtypes in the human brain (Bowers et al., 2012). NPY is co-localized with GABA within the BLA (Bowers et al., 2012). Since NPY also contributes to resilience after stress (Feder et al., 2009; McGuire et al., 2011) it might have an interesting role in regulating fear acquisition and extinction under stressful conditions.

*Animals:* intracerebroventricular NPY resulted in an improvement of extinction acquisition in fear-potentiated startle (Gutman et al., 2008), while mice lacking both, Y1 and Y2 receptors, showed accelerated acquisition of conditioned fear, excessive recall of conditioned fear and impaired fear extinction (acquisition and recall; Verma et al., 2012). Over-expression of the selective Y2-agonist NPY_3–36_ in the CE of the amygdala resulted in improved extinction acquisition in cued (not contextual) fear conditioning and minor return of fear (less spontaneous recovery and reinstatement), while local destruction of Y2-receptors impaired extinction memory (Verma et al., 2015).

*Humans:* we are not aware of data addressing NPY in fear extinction studies with healthy humans. Indirect evidence comes from data revealing that subjects with genotypes that predispose to have low NPY levels showed a higher responsiveness to aversive stimuli (negative words) in the mPFC and rostral ACC than those with intermediate or high expression (Mickey et al., 2011). Further, patients with PTSD (Rasmusson et al., 2000) were shown to have lower NPY baseline levels than healthy controls.

*In sum:* first animal data converge that NPY might help to improve fear extinction acquisition and recall. The results in humans only come from quasi-experimental and correlative studies in patients, but provide a first hint that high NPY levels might also be relevant for improving fear extinction in humans.

*Oxytocin.* Among other locations, oxytocin receptors are also located in the amygdala and the dorsal and ventral hippocampus. Moreover, due to its regulatory effects on the HPA-axis activation, oxytocin is an interesting neuropeptide for fear extinction under stress.

*Animals:* oxytocin was shown to inhibit excitatory output to brain-stem fear-expression centers when injected into the CE of the amygdala, here interacting with vasopressin (Huber et al., 2005). It also resulted in higher heart rate variability (as an indicator of a more vagal tone of heart rate modulation) during extinction acquisition and reduced expression of fear (Viviani et al., 2011). Thus, one should expect oxytocin to improve extinction recall, but the time-point of administration appears to be very important.

Intracerebroventricular injection of synthetic oxytocin *prior to fear acquisition* did not affect fear acquisition but facilitated extinction acquisition and retrieval, an effect that was abolished by an oxytocin-receptor antagonist. Interestingly, oxytocin given *prior to extinction acquisition* instead impaired extinction learning; this effect was again abolished by blockade of the oxytocin receptor (Toth et al., 2012). This suggests, that oxytocin needs to be already administered prior to fear acquisition, and that caution is necessary when considering the use of oxytocin after the acquisition of a trauma. Recently, oxytocin was shown to reduce reconsolidation of fear memories in rats after a single systemic injection after the reactivation of fear (Hou et al., 2015).

*Humans*: there is some evidence for facilitating effects of pre-extinction oxytocin on subsequent extinction acquisition (Eckstein et al., 2015) and fear extinction recall (Acheson et al., 2013) while a pilot clinical study using oxytocin prior to an exposure session in spider phobia revealed even debilitating effects (Acheson et al., 2015).

In a fear-potentiated startle paradigm (Acheson et al., 2013), subjects received either intranasal oxytocin or placebo *after fear acquisition*, followed by extinction acquisition (45 min after substance administration) on day 1, and extinction recall on day 2 (24 h later). While the oxytocin group showed higher startle magnitudes in the early extinction trials, both groups manifested reduced responding by the end of extinction acquisition on day 1. On day 2, extinction recall was better after previous oxytocin treatment, suggesting an oxytocin-induced enhancement of extinction consolidation. Eckstein et al. (2015) only addressed fear acquisition and extinction acquisition on a single day in a differential conditioning paradigm. They measured SCR and blood oxygen dependent (BOLD) signal from functional magnetic resonance imaging (fMRI). Intranasal oxytocin (vs. placebo) was again given after fear acquisition. In the early trials of extinction acquisition, oxytocin (vs. placebo) even induced a higher SCR, but also a higher activity in the right PFC. By the end of extinction, oxytocin resulted in lower SCRs. Moreover, oxytocin led to an unspecific inhibition (affecting CS+ and CS−) in amygdalar responses in early and late extinction acquisition. Extinction recall was not assessed here.

Patients: in a recent study, intranasal oxytocin (vs. placebo) was delivered as a pretreatment immediately prior to a single-session exposure treatment for spider phobia (Acheson et al., 2015). Dependent measures covered self-report of spider-phobia symptoms, phobic avoidance behavior, and trust into treatment (treatment creditability and therapeutic alliance). Follow-up was located 1 week and 1 month after exposure. Oxytocin- as compared to placebo-treated subjects manifested even higher clinical ratings of fear symptoms, higher avoidance behavior (as rated by clinicians) and less confidence into the treatment and a trend for less therapeutic alliance.

*In sum:* data suggest that some caution is necessary when delivering oxytocin prior to extinction (or prior to exposure therapy). There is evidence for both, extinction-improving and extinction-debilitating effects. More data are needed to find out whether oxytocin might especially affect extinction consolidation (and not extinction acquisition) and how oxytocin acts on extinction neurocircuits.

#### Second-Wave Mediators

##### GCs, extinction learning and the role of circadian variation/rhythm

There is a substantial amount of evidence suggesting that GCs have a positive influence on fear extinction learning and memory. Systemic (Cai et al., 2006; Yang et al., 2006, 2007; Brinks et al., 2009; Blundell et al., 2011) and intra-amygdala administration (Yang et al., 2006) of GC agonists (e.g., corticosterone, dexamethasone) facilitates fear extinction memory when given prior to or directly after extinction training. In contrast, GR antagonists or the GC-synthesis inhibitor metyrapone impair extinction memory when administered systemically (Barrett and Gonzalez-Lima, 2004; Yang et al., 2006, 2007; Blundell et al., 2011) or into the amygdala (Yang et al., 2006). Importantly, the facilitating effect of GR-agonist dexamethasone was blocked by intra-amygdala infusion of the GR-antagonist mifepristone (Yang et al., 2006), suggesting that GRs in the amygdala are mediating the enhancement of extinction. Comparing post-reactivation administration of GC or a β-blocker (Abrari et al., 2008) suggest that GCs affect extinction memory *per se*, without compromising reconsolidation. Yet, GCs may have opposite effects on fear extinction mediated by different actions on GRs and MRs. Systemic administration of a low-dose GR agonist (dexamethasone) or administration of the MR-antagonist spironolactone both enhanced contextual fear extinction (Ninomiya et al., 2010), whereas MR-agonist fludrocortisone impaired extinction. High-dose dexamethasone or the GR-antagonist mifepristone had no effect (Ninomiya et al., 2010).

Importantly, data also suggest, that GRs can alleviate stress-induced extinction impairments. In the immobilization stress model of PTSD stress exposure impairs fear extinction a week later (Sawamura et al., 2015). However, systemic GR-agonist dexamethasone 4 h before extinction rescued extinction deficits in previously stressed rats and improved extinction acquisition and recall (Sawamura et al., 2015). Conversely, in the single prolonged stress (SPS) model, GC-synthesis inhibitor metyrapone exacerbated SPS-induced extinction recall deficits (Keller et al., 2015b). GR-signaling in the hippocampus has also been recently implicated to be one of the pathways mediating the enhancement of contextual fear extinction by novelty (Liu et al., 2015).

In sum, animal studies show that endogenous GCs play an important part in successful fear extinction and extinction memory, and GC administration can improve extinction. These effects are probably mediated through central GRs, especially in the amygdala and hippocampus.

*Humans:* studies addressing the effects of GC administration on fear extinction in humans are scarce and results are mixed. While there are reports that hydrocortisone (30 mg) administration impaired extinction learning in men (SCR and BOLD; Merz et al., 2014b), other studies find an enhancement in (BOLD-signal) differentiation during extinction in women using hormonal contraceptives (Tabbert et al., 2010). Here however (Tabbert et al., 2010), hydrocortisone increased responses to the CS− (never paired with the US) vs. CS+ (previously paired with the US) which is hard to interpret. Another study found no effect of the same dose of hydrocortisone (30 mg) in men and free-cycling women (Merz et al., 2012a). On the other hand, clinical studies have shown enhanced outcome of exposure therapy, when GCs were administered prior to exposure training for specific phobias (Soravia et al., 2006, 2014; de Quervain et al., 2011). For PTSD, combining exposure techniques with GCs led to lower avoidance/numbing symptoms compared to placebo 1 week after intervention, but the effects did not last at a 1 month follow-up (Surís et al., 2010). Low-dose cortisol alone has also shown some promising results in a pilot study with 3 PTSD-patients (Aerni et al., 2004). Endogenous levels of GCs show a marked diurnal variation with a peak in the early morning after awakening. This peak has been linked to enhanced extinction/extinction recall (Pace-Schott et al., 2013) and a better outcome of exposure therapy in humans in the morning (Lass-Hennemann and Michael, 2014). However, only one study shows a circadian effect with a direct positive association between endogenous GC levels and exposure therapy outcome (Meuret et al., 2015).

*To summarize:* in line with animal studies, in humans, GC administration combined with exposure therapy has shown some promise to yield a better therapy outcome. Similar conclusions can be drawn from diurnal effects in humans. Both lines of evidence indirectly point to a possible enhancement of fear extinction by GCs in humans, as repeatedly shown in rodents. However, the few studies with GC administration directly examining fear extinction in healthy volunteers cannot back up clinical trials yet.

### Insights from Stress-Induction Studies Triggering the First and Second Wave of the Stress Response

So far, we reported effects of single mediators of the stress response from pharmacological manipulations. This is important, but no substitute for experimentally induced stress. As stress responses are typically defined by the interplay of multiple mediators (Joëls and Baram, 2009), and as GC effects in the amygdala were shown to depend on arousal-induced NA-signaling (Roozendaal et al., 2006a), single pharmacological manipulations might be insufficient. Despite the potential relevance for clinical settings, especially human studies on the effects of acute stress on fear extinction are still rare (for a review, see Raio and Phelps, 2015). Below, we review animal and human stress-induction studies on fear extinction focusing broadly on acute stress effects. Effects of chronic stress on fear extinction and effects of stress during pre- and postnatal development on extinction are beyond the scope of this review.

*Animals:* the field has focused on designs where extinction follows one to several days after stress, thus reflecting longer lasting effects of second-wave mediators. A number of procedures have been suggested to model features of PTSD in laboratory animals (e.g., Rau et al., 2005). The SPS model is especially well studied with fear extinction. SPS comprises 2 h restraint stress, 20 min of forced swim stress, and exposure to ether until general anesthesia (Yamamoto et al., 2008). When applied 7 days before fear acquisition and extinction the SPS was repeatedly shown to impair extinction learning and especially extinction recall while leaving fear acquisition intact (Yamamoto et al., 2008, 2009; Knox et al., 2012a,b; Ganon-Elazar and Akirav, 2013; Matsumoto et al., 2013). The delayed effects of SPS on fear extinction were linked to enhanced GR-expression in the hippocampus and PFC (Knox et al., 2012b) and to upregulation of NMDA-receptor mRNA in the hippocampus (Yamamoto et al., 2008; Matsumoto et al., 2013). However, no impairment of extinction was found after a shorter time interval between stress and learning (Knox et al., 2012a), or after reducing stressor intensity by omitting either one of the SPS components (Knox et al., 2012b). In a different model, placing repeated forced swim stress (10 min/day for 3 days) 24 h before cued fear acquisition and 48 h before extinction also impaired extinction learning (but not fear acquisition) and produced dendritic retraction in IL neurons in the PFC (Izquierdo et al., 2006).

Behavior during/after extinction can be seen as the result of a competition between the original fear memory and the new inhibitory extinction memory. Therefore, placing stress before fear acquisition makes precise inferences about stress effects on extinction difficult, as stress could have modified the original fear memory. However, there is also evidence that stress impairs fear extinction even when animals are stressed after fear acquisition: 30-min of elevated platform stress 24 h before extinction impaired extinction learning and was associated with changes in BLA morphology, including dendritic retraction and debranching but also increased spine density (Akirav et al., 2009; Maroun et al., 2013).

In some cases, stress exposure can also facilitate fear extinction. A single exposure to 3 h of immobilization stress 14 days (but not 2 days) before contextual fear acquisition and extinction significantly improved extinction recall (Kirby et al., 2013). The stress effects were linked to increased neurogenesis in the dorsal hippocampus and seem to be mediated through stress-induced GCs activating basic fibroblast growth factor (FGF2; Kirby et al., 2013).

*In sum:* the combined evidence suggests that stress impairs the consolidation/recall of fear extinction. The impairment requires time (>24 h) and is associated with structural changes in extinction-relevant structures including dendritic morphology, and receptor density. Stress-induced extinction impairments seem more likely with severe, prolonged and high-intensity stressors.

*Humans:* here, we group studies by (a) the type of stressor and its validity for triggering the first- vs. second-wave stress response and (b) by considering the timing of the stressor relative to stages of extinction memory formation (encoding, consolidation, recall). Stress placed already before fear acquisition will give a better comparison to the animal studies above. Stress placed after fear acquisition but before extinction gives insights into effects on encoding. Stress after extinction learning informs us about consolidation effects. Finally, stress before extinction recall captures influences on recall independent from learning and consolidation.

Although considerably fewer in number, human studies also suggest that stress placed *before fear acquisition* (and thus also extinction) may impair fear extinction. We have recently shown (Antov et al., 2013), that inducing the first-wave stress response immediately before fear acquisition impaired extinction even without a second-wave GC-increase. We used the CPT, where participants immerse their hand into ice-cold water for a max of 3 min and found increased extinction resistance compared to control, probably due to a strengthening of the original fear memory. Yet, a psychosocial stressor inducing significant GC-increases had no effects on conditioning (Antov et al., 2013). In contrast, a psychosocial stressor 70 min before fear acquisition impaired extinction (Jackson et al., 2006), and uncontrollable shocks 7 days before fear acquisition reduced extinction recall (Hartley et al., 2014).

Placing first-wave stress (CPT) *after acquisition* but immediately *before extinction* training facilitated extinction acquisition and extinction recall without second-wave GC-increases (Antov et al., 2015). Bentz et al. (2013) also placed the CPT prior to extinction training: they found that CPT impaired recall of the original fear memory (measured in subjective US expectancy) in men but not in women. As discussed by the authors (Bentz et al., 2013), this study has some important limitations precluding inferences about stress effects on extinction: (1) there was no evidence for fear learning (or extinction) in physiological conditioning measures (SCR, heart rate), (2) there was also no extinction learning in any measure. A variant of the CPT including social evaluation (SECPT, Schwabe et al., 2008), also inducing GC-increases, placed *after fear extinction* impaired extinction recall 24 h later, especially when tested in the acquisition context (Hamacher-Dang et al., 2015). Finally, two studies suggest that stress also affects the retrieval of fear extinction: CPT associated with GC-increases 15 min *before delayed extinction recall* (Raio et al., 2014) significantly impaired recall performance. Conversely, the SECPT placed 20 min prior to extinction recall and 24 h after extinction learning, reduced contextual fear renewal (Merz et al., 2014a), probably indicative of enhanced extinction recall.

*In sum:* the few available human studies yield mixed results, but there is some evidence that first-wave stress before extinction learning improves extinction acquisition and recall whereas second-wave stress before recall testing impairs extinction recall.

## Sex, Sex Hormones and Fear Extinction Under Stress

### Sex, Sex Hormones and Fear Extinction

Compared to men, the prevalence of anxiety (Kessler et al., 2005; Somers et al., 2006; Eaton et al., 2012) and trauma- and stressor-related disorders (Kessler et al., 1995; Tolin and Foa, 2006; Kilpatrick et al., 2013; Zoladz and Diamond, 2013) is up to two times higher in women.

Levels of circulating gonadal hormones (mainly estrogens) are regarded to contribute to these sex differences in psychopathology (Lebron-Milad and Milad, 2012; Cover et al., 2014).

Estrogens are a class of steroidal sex hormones that include estrone (E1), estradiol (E2), estriol (E3), and estetrol (E4). E2 is produced by the ovaries and (to a smaller amount) by the adrenal cortex, and by the testes in men by conversion from testosterone via the enzyme aromatase or by conversion of androstenedione to E1 and conversion of E1 to E2. E1 is the dominant estrogen during menopause and E3 and E4 dominate during pregnancy. E2 is the predominating estrogen in non-pregnant women in their reproductive years when E2-levels are considerably higher in women than in men. In humans, an idealized menstrual cycle lasts 28 days and includes the following phases of different concentrations of E2 and P4 (Becker et al., 2005): E2 and P4 are low during the early follicular phase (approx. cycle days 1–8). During the late follicular phase, P4 remains low, while E2 rises to reach its peak immediately before ovulation (midcycle, approx. days 13–14). Peak P4 levels are only reached during the mid-luteal phase, where there is also a second less prominent peak in E2. In rodents, the estrous cycle lasts 4–5 days and covers four phases: metestrus (low E2 and low P4), diestrus (moderate P4 and low E2), proestrus (high E2 and high P4), and estrus (low E2 and low P4; Maeng and Milad, 2015).

E2 exerts its effects via two types of steroidal estrogen receptors located in the cell nucleus or the cytoplasm: estrogen receptor alpha (ERα) and beta (ERβ) which are both widely distributed across the brain (Gillies and McArthur, 2010). ERα are broadly distributed across cortical and subcortical structures including hippocampus, amygdala, hypothalamus, and brain stem, whereas ERβ distribution is less wide-spread with medium to high ERβ density throughout the cortex including the vmPFC, high density in the hippocampus, and in some hypothalamic nuclei (Gillies and McArthur, 2010; Cover et al., 2014). Importantly, ERs are well expressed in brain regions critical for fear acquisition and extinction including amygdala subnuclei, hippocampus and vmPFC (Shughrue et al., 1997; Österlund et al., 1998, 2000; Zhang et al., 2002; Weiser et al., 2008).

Neuroimaging studies reveal that circulating levels of E2 in women affect the reactivity of brain structures involved in both, stress and fear acquisition and extinction. Specifically, women scanned during the midcycle phase (peak E2, but low P4) exhibited significantly less responses to high arousing, aversive visual stimuli (International Affective Picture System, IAPS; Lang et al., 2008) in the amygdala, ACC, orbitofrontal cortex, mPFC, hippocampus, PAG, and several hypothalamic nuclei compared to both, men and women tested during the early follicular phase (low E2 and low P4; Goldstein et al., 2005, 2010).

Estrogens were shown to be especially important in fear extinction: in an elegant series of experiments in rats, Chang et al. (2009) found faster extinction of contextual fear conditioning in female than male rats, especially when in the high E2-phase of the estrous cycle (proestrus). The authors also observed enhanced extinction after systemic and central E2-administration to ovariectomized animals and traced the effects back to hippocampal ERβ by using selective ERα/ERβ agonists.

There is accumulating evidence for the role of E2 especially for *extinction recall* in both laboratory animals and healthy humans: low E2-levels were shown to impair and high E2-levels to enhance extinction recall, typically tested 24 h after extinction training (Milad et al., 2006, 2009a, 2010; Zeidan et al., 2011; Graham and Milad, 2013). Studies also include data on low-level estrogen under contraceptives (Graham and Milad, 2013). Moreover, in mice both, high endogenous E2 and activation of ERβ, enhanced glutamatergic transmission and synaptic plasticity in the IL mPFC (Galvin and Ninan, 2014), a structure associated with consolidation and recall of extinction. Interestingly, inhibition of estradiol synthesis by an aromatase inhibitor (fadrozole) in male rats, significantly impaired their extinction recall (Graham and Milad, 2014). Adequate controls (such as delivering a single dose of E2 after previous aromatase inhibitor) gave first evidence that fear extinction in males may also depend on acute effects of estrogen synthesized *de novo* and acting presumably via non-genomic mechanisms. In contrast to the majority of studies supporting an enhancement of extinction by high E2-levels, there is recent evidence for the opposite effect, i.e., enhanced fear memory and impaired extinction memory under high E2-levels (McDermott et al., 2015). But this study did not assess female rats under natural E2 conditions but studied E2 replacement in female ovariectomized mice (vs. mice after sham surgery) instead. Chronic high-dose, not low-dose, E2 administration in pre-puberty (4 weeks) and in adult age (10 weeks), impaired extinction but only in contextual, not cued, fear conditioning. This suggests an adverse effect of chronic high E2-levels for hippocampus-mediated fear inhibition. Anyway, we have to take into account, that chronic high E2 actions after ovariectomy do not necessarily reflect “normal” E2 actions within the brain and periphery.

Addressing brain correlates during fear extinction and considering different E2-levels in humans revealed that women using oral contraceptives (having suppressed E2-levels) showed higher differential BOLD responses to the CS+ (previously paired with the US) during fear extinction compared to men and women in the luteal phase of the menstrual cycle (high P4, medium-high E2) in the amygdala, ACC, and vmPFC (Merz et al., 2012a). This is in line with impaired extinction when E2-levels are low. The study also included administering cortisol vs. placebo but this did not affect the results (see also “Second-wave mediators” Section). Accordingly, a recent study examining women during the early follicular and luteal phases of the menstrual cycle (Wegerer et al., 2014) reported that low E2 but not P4 was associated with poorer extinction and with higher intrusive memories.

Patients: with PTSD exhibit marked fear extinction deficits as compared to healthy controls (Milad et al., 2009b; Inslicht et al., 2013) or trauma-exposed and non-trauma-exposed healthy controls (Milad et al., 2009b). Interestingly, fear inhibition/extinction deficits have been linked to low levels of E2 in both female PTSD-patients (Glover et al., 2012) as well as in healthy and traumatized women (Glover et al., 2013). A recent study by Shvil et al. (2014), addressing sex differences in trauma-exposed healthy controls vs. PTSD-patients, found impaired extinction recall (higher SCRs to the extinguished CS+) and higher activity in the left rostral dACC in men as compared to women within the PTSD group, but not in trauma-exposed healthy controls. The result also suggests that interventions improving fear extinction are highly important in male patients with PTSD.

*In sum:* the available data suggest an important role of E2 for fear extinction with high E2-levels enhancing extinction acquisition and especially extinction recall (with most of the studies comparing different E2-levels within females). The assessment of E2-levels should be thus included in fear extinction studies. Comparing women in different cycle phases and under different natural E2-levels is only a quasi-experimental approach. Thus, experimental studies would be important with experimental variation of sex-hormone levels.

### Interaction of Stress and Sex Hormones in Fear Extinction

Although trauma- and stressor-related disorders are associated with deficits in fear extinction, we still do not know much about the effects of *acute stress* on fear acquisition and extinction in healthy humans (Raio and Phelps, 2015). Importantly, animal data suggests that stress effects on fear conditioning are sex specific (Dalla and Shors, 2009). For example, in ovariectomized female rats an injection of E2 (45 μg/kg) was able to alleviate conditioned fear responses after SPS (Mirshekar et al., 2013), suggesting an E2 × stress interaction. In intact females (as compared to males), SPS did not impair extinction learning or recall, regardless of estrous cycle phase (Keller et al., 2015a).

In humans, Zorawski et al. (2005) found that the level of post-acquisition GCs was positively correlated with fear acquisition performance (SCR) in men, but not in women. This was later replicated (Zorawski et al., 2006) with an added stressor after acquisition to achieve higher GC-levels. Again, post-acquisition GCs were only correlated with acquisition in men, but not in women. This suggested a sex-specific relationship between GCs and fear acquisition. Similarly, Merz et al. (2013a) report a positive correlation between basal cortisol levels and amygdala activity during fear acquisition only in men and in women taking oral contraceptives, but not in women in their luteal cycle phase. In a first experimental study (Jackson et al., 2006) reported that psychosocial stress (inducing cortisol increases) enhanced differential SCRs during both acquisition and immediate extinction in men, but had no effect in women. However, subsequent neuroimaging studies report the opposite effect after a psychosocial stressor: reduced SCRs and BOLD responses in men during acquisition and enhanced BOLD responses after stress in women (Merz et al., 2013b). Similar results, with higher conditioned BOLD responses in women, and impaired responses in men were reported from the same group (Stark et al., 2006; Merz et al., 2010) for fear acquisition after administration of cortisol (oral, 30 mg hydrocortisone) vs. placebo.

Cortisol effects on BOLD responses during fear acquisition in women were shown to depend on gonadal hormone availability: women using oral contraceptives showed enhanced conditioned BOLD responses after cortisol, whereas cortisol impaired responding in men and free-cycling women in their early follicular (low E2, low P4) and mid-luteal (low to mid E2, high P4) cycle phases (Merz et al., 2012b). But in another study of that group, comparing men and women in their luteal phase and women taking contraceptives, the additional experimental factor of administering cortisol (vs. placebo) had no effect on extinction acquisition (Merz et al., 2012a). In a recent study of our group (Antov and Stockhorst, 2014), we tested the effect of a psychosocial stressor that preceded fear acquisition. Fear acquisition started in the maximum of the peripheral cortisol peak. We examined men and free-cycling women either in the early follicular phase (low E2, low P4) or in the mid-cycle phase (high E2, low P4) and tested fear acquisition, extinction acquisition (day 1) and extinction recall (day 2). We found an interaction between stress exposure and natural E2-status within women: in mid-cycle women, extinction recall was better when fear acquisition had been preceded by stress, whereas the inverse was true in early follicular women. Thus, extinction recall of conditioned fear acquired after stress depends on estrogen status in women. Consequently, we suggest that for extinction-based exposure therapy in women, cycle phase and/or E2-level should be taken into account. We assume that the mid-cycle phase (with high E2- and low P4-levels) provides an adequate phase to re-expose women to the aversive CSs during exposure therapy. In healthy volunteers, exposure to aversive stimuli during this high E2-cycle phase was also associated with less intrusive memories (Wegerer et al., 2014).

Considering the higher prevalence of anxiety, trauma- and stressor-related disorders in women, and extinction deficits under low as compared to high E2-levels (within women), one might wonder why low levels of E2 are disadvantageous for women, but (often) not for men who also have low levels of circulating E2. Most importantly, one has to keep in mind that testosterone is rapidly converted to estrogen in the brain via aromatase. Thus, men do take advantage of estradiol actions within the brain, although they have low levels of circulating E2. This is supported by data showing that the inhibition of estradiol synthesis by an aromatase inhibitor in male rats significantly impaired extinction (Graham and Milad, 2014). When it comes to the interaction between female sex hormones and stress in fear conditioning studies with humans, there is some initial evidence that there might be a higher resemblance in the response pattern of men and naturally cycling women in a high estrogen status than with women in a low estrogen status (Antov and Stockhorst, 2014), or than with women using contraceptives (Merz et al., 2012b). Here—in addition to the above argument—one might also consider differences in binding capacities of MRs and GRs for corticosterone, or cortisol, respectively. These binding capacities were shown to differ between males and females, and—within females—also between different cycle phases (Ter Horst et al., 2012). But, most importantly, much more studies are needed to assess the interaction between stress and sex, or sex hormones, especially with regard to extinction.

### A Role of Male Sex Hormones and of Hormonal Transitions in Fear Extinction

While fear extinction studies measuring female sex hormone levels are already sparse, there are hardly any studies addressing the role of *male sex hormones* for fear extinction. Moreover, these remaining data are mainly based on studies using castration, not exogenous administration of androgens.

In an early study (Anagnostaras et al., 1998), adult male rats (age 12–17 weeks) were either castrated or sham-operated or remained intact. Groups did not differ in their extinction acquisition of contextual fear, or in hippocampal long-term potentiation. Different results were obtained by McDermott et al. (2012): castration during prepuberty (4 weeks old) as well as thereafter (10-weeks old) resulted in an impairment of extinction in a contextual conditioning paradigm, not in cued conditioning, suggesting that testosterone might act to improve fear extinction in hippocampal-dependent, not in amygdala-mediated fear memory. In a recent fear conditioning study with healthy men (Pace-Schott et al., 2013), testosterone levels were also measured. Interestingly, a higher testosterone/cortisol (T/C) ratio predicted better extinction acquisition, but only in the morning hours (i.e., when endogenous cortisol levels are high). The T/C ratio (not the single hormones) was negatively correlated with the remaining CR (SCRs). Although these data are only correlative, they encourage examining the role of testosterone on fear extinction under stressful conditions. Experimental studies are of interest where testosterone is manipulated under stressful vs. control conditions. In the meantime, it would be helpful to measure testosterone levels in fear conditioning studies.

Another approach to study the role of sex hormones for fear-related memory and extinction memory is to examine developmental phases of larger hormonal changes. Again, data are sparse. There is some evidence (recently reviewed by Baker et al., 2014) for impaired fear extinction in adolescents as compared to both, younger individuals as well as adults. One explanation refers to functional changes in the connectivity between prefrontal cortex and amygdala during this developmental stage. Moreover, adolescence is a stage of high vulnerability to stress (Lupien et al., 2009).

For women, pregnancy and perimenopausal stages are additional phases of larger hormonal changes. For example, there is a heightened risk for depression when E2 production is sharply reduced after menopause (for an overview, see Cover et al., 2014) and there is evidence, that estradiol replacement can improve depressive symptoms in women with a history of perimenopausal depression (e.g., Schmidt et al., 2015). More studies specifically addressing fear extinction during developmental stages and hormonal transitions are necessary. This might also include conditions where estrogen receptors are blocked. This is the case in the pharmacological adjunct therapy in estrogen-receptor positive breast cancer patients receiving the estrogen-receptor blocker tamoxifen.

## Conclusion

Our review aimed at elucidating how single mediators of the stress response (hormones, neurotransmitters, and neuropeptides) and the entire natural stress response (covering the sequence of the first and the second wave of the stress response) contribute to fear extinction. Our stress approach is guided by the idea that classical stress-response mediators are also agents within the fear macro- and microcircuits. Thus, explicitly investigating stress or stress-response mediators in fear acquisition and fear extinction might mimic the natural situation of acquiring anxiety, trauma- and stressor-related disorders and of being re-exposed to the fear reminders during extinction-based exposure therapy.

Concerning the mediators of the first-wave stress response (monoamines, opioids, eCBs), animal data strongly suggest that rapidly acting NA enhances extinction learning and extinction memory processes; for humans, data are less conclusive and more human studies on NA effects in extinction are warranted. Dopaminergic as well as serotonergic signaling improves extinction memory in animals and humans, while a number of modifying conditions (dose, temporal spacing, targeted receptor-subtypes, chronic vs. acute administration, gene polymorphisms) have to be considered. An improvement of extinction also occurs under opioid signaling and eCBs, with first evidence also in humans. Endogenous GCs play an important part in successful fear extinction and extinction memory. In animal studies, data converge that GC administration can improve extinction when the learning task is in “sync” with the cortisol peak thus improving consolidation of extinction memory. In line with animal studies, in humans GC administration combined with exposure therapy has shown some promise to yield a better therapy outcome. Similar conclusions can be drawn from diurnal effects in humans. However, the few studies with GCs administration directly examining fear extinction in healthy volunteers cannot back up clinical trials yet.

Considering the natural stress response, animal studies suggest that stress impairs the consolidation/recall of fear extinction. The impairment requires time (>24 h) and is associated with structural changes in extinction-relevant structures including dendritic morphology, and receptor density. While results are mixed in humans, there is some evidence that extinction acquisition during the first-wave stress response (e.g., after the CPT) improves extinction recall. On the other hand, second-wave stress before testing impairs extinction recall.

While the prevalence of anxiety, trauma- and stressor-related disorders is up to two times higher in women than in men, studies addressing the level of E2 reveal better fear extinction, especially fear extinction recall, and higher vmPFC-activity, in cycle phases of a high as compared to low E2-level in women. On the other hand, in PTSD-patients, behavioral fear-extinction deficits as well as reduced vmPFC activity have been linked to low E2-levels. There is first evidence for an interaction between stress exposure and natural E2-status within women: in mid-cycle women, extinction recall was better when fear acquisition had been preceded by stress, whereas the inverse was true in early follicular women. Thus, extinction recall of conditioned fear acquired after stress depends on estrogen status in women. Consequently, we suggest that for extinction-based exposure therapy in women, cycle phase and/or E2-level should be taken into account. In case of low E2-levels, drugs increasing dopamine and serotonin levels might improve the success of extinction, or exposure therapy.

We predict that behavioral and pharmacological interventions using the mediators of the first-wave stress response applied prior to *extinction acquisition* should enhance extinction memory. For GC-related interventions (being related to the second wave of the stress response), we should consider that the consolidation phase of the conditioning task (i.e., extinction acquisition) is in “sync” with the GC peak in order to take advantage of extinction-improving GC effects whereas high GC levels at retrieval are expected to impair extinction memory retrieval.

Since exposure therapy can be described as a form of extinction learning, the presented data thus might also allow predicting which of the above pharmacological interventions and behavioral types of stress induction could constitute adjuncts to exposure therapy. Concretely, DA- and 5-HT-reuptake inhibitors, endorphins, eCBs, and GCs prior to extinction (in “sync” with the consolidation phase) as well as stressors strongly affecting the first-wave stress response (such as the CPT increasing the noradrenergic tone) could be successful. Moreover, data presented here also encourage taking cycle phase and/or contraceptive use into account when scheduling exposure sessions for women.

## Author Contributions

Both authors, US and MIA, fulfill the four criteria for authorship and contributed equally to the present manuscript.

## Conflict of Interest Statement

The authors declare that the research was conducted in the absence of any commercial or financial relationships that could be construed as a potential conflict of interest.
